# Transcutaneous Bilirubin Measurements Can Be Used to Measure Bilirubin Levels during Phototherapy

**DOI:** 10.1155/2018/4856390

**Published:** 2018-03-20

**Authors:** Saad Abdullah Alsaedi

**Affiliations:** Department of Pediatrics, Faculty of Medicine, King Abdulaziz University, Jeddah, Saudi Arabia

## Abstract

**Objective:**

To determine whether transcutaneous bilirubin measurements (TcB) before and during phototherapy taken from covered skin during phototherapy correlate with total serum bilirubin (TSB) levels.

**Study Design:**

In this prospective observational study, healthy term newborns who required TSB measurements were included. TcB measurements were taken from the forehead before starting and during phototherapy using the BiliChek device. Before starting phototherapy, part of the forehead was covered. Blood for TSB measurement was collected within 5 minutes of TcB measurements. Correlations and mean differences between TcB and TSB before and during phototherapy were calculated.

**Result:**

Paired TSB and TcB measurements before and during phototherapy in 151 newborns were performed. The mean gestational age was 38.8 weeks and birth weight was 3.1 kg; 53% were male. Before starting phototherapy, TSB and TcB were 183.8 ± 41.6 and 190.5 ± 43 *μ*mol/l, respectively. During phototherapy, TSB and TcB were 191.8 ± 39.4 and 187.8 ± 45.3 *μ*mol/l, respectively. Linear regression analysis showed a significant correlation between TcB and TSB before starting phototherapy and during phototherapy (*r*: 0.85; *p* < 0.001 and *r*: 80.0; *p* < 0.001), respectively. Before starting phototherapy, the mean difference between TSB and TcB was 6.2 ± 23.2 *μ*mol/l, with a 95% CI of −39.3 to 51.7 *μ*mol. During phototherapy, the mean difference was −2.8 ± 23.5 *μ*mol/l, with a 95% CI of −48.9 to 43.3 *μ*mol/l.

**Conclusion:**

TcB measurements from covered skin in jaundiced term infants during phototherapy correlate with TSB and can be used to monitor bilirubin levels during phototherapy.

## 1. Introduction

Hyperbilirubinemia is common during the neonatal period and sometime requires treatment with either phototherapy or exchange transfusion. Management of neonatal hyperbilirubinemia becomes more challenging when at-risk newborn infants are discharged early without appropriate postdischarge follow-up. This increases the risk of severe hyperbilirubinemia and its complications such as acute bilirubin encephalopathy, and bilirubin-induced neurologic dysfunction (BIND) in these infants [1]. Although the measurement of TSB is still the standard of care in the assessment of neonatal hyperbilirubinemia, it requires venous or heel prick blood samples, which is an invasive and painful procedure. Transcutaneous bilirubin (TcB) measurement is an easy, painless, and timesaving alternative to TSB measurement [2, 3]. Several studies have shown a good correlation between TSB and TcB measurements in term and late preterm infants [3, 4]. TcB measurement is an essential part of screening for neonatal jaundice that can decrease the need for TSB [4, 5]. Jaundiced newborn infants under phototherapy require close monitoring of TSB, which means frequent blood sampling. In addition, there has been an increasing trend towards home phototherapy in the low-risk term and near-term infants, where follow-up assessments of TSB are cumbersome [6–8]. Such infants can benefit from TcB measurement at home if validated. Bilirubin in the skin exposed to phototherapy is modified and significantly affects TcB. Phototherapy converts the bilirubin to the more water-soluble lumirubin, which is excreted by the body. This process causes blanching of the skin, which obviously will change TcB levels. However, the accuracy of TcB measurement during phototherapy is still not clear. Some studies reported that, by covering the skin during phototherapy, more accurate approximations of TSB could be made with TcB measurement though TcB measurement tends to underestimate TSB when bilirubin levels are >214 *μ*mol/L [6]. However, other studies reported insignificant difference between TcB and TSB measurements [9, 10]. The sample size of some of these studies was small and the study populations were not homogeneous where both term and preterm newborns were included. The aim of this study was to evaluate the accuracy of TcB measurement from a shielded area at the forehead of jaundiced term newborns during treatment with phototherapy.

## 2. Methods

### 2.1. Subjects

This was a prospective observational study conducted at the newborn nursery of a tertiary care center with 4000 deliveries per year during the period of January 2015 to December 2016. We conducted the study according to the principles of Helsinki Declaration. The Institutional Review Board approved the study. We obtained informed consent from the parents of the newborn infants before inclusion in the study. We included healthy term newborns who required phototherapy for management of neonatal hyperbilirubinemia as per the decision of the treating physician. We excluded infants who had conjugated hyperbilirubinemia, major congenital malformation, sepsis, or congenital viral infections.

### 2.2. Protocol

During the study period, we measured TcB in all jaundiced, otherwise healthy, term newborns who required TSB measurements. The attending neonatologist made the decision when to start continuous phototherapy based on the American Academy of Pediatrics guideline [5] as well as the frequency of obtaining TSB measurements. In our nursery, we use a standard phototherapy unit (Photo Therapy 4000, Draeger Medical Telford, PA, USA). The total power irradiance during phototherapy was 28–30 W/cm^2^/nm. We included in this study healthy term newborns who required phototherapy. Both eyes were protected from phototherapy light with eye patches (Biliband, Natus Medical Incorporated, Seattle, WA, USA). We measured TcB before and during phototherapy from the same area in the middle of the forehead. We avoided areas with hair, hyperpigmentation, bruises, and hemangioma. Before starting phototherapy, we covered a portion at the middle of the forehead, by a photo-opaque patch 2.5 cm in diameter (BilEclipse Phototherapy Patch, Respironics, Murrysville, PA, USA) to shield it from light exposure. We used a single BiliChek® device to measure TcB. We measured all TcB from the covered part of the forehead, while the infant is under phototherapy within 5 minutes of drawing blood for TSB measurements. Once phototherapy was started, we repeated the TcB measurement with the first TSB. We turned off the phototherapy during TcB measurements and blood sampling for TSB. We calibrated the BiliChek before each measurement using a disposable probe (BiliCal, SpectRx, Norcross, GA, USA) as per manufacturer's instruction [11]. BiliChek displays the average bilirubin in micromole/l of 5 measurements for each TcB. BiliChek was in use in our nursery to measure TcB for the last 5 years, so all the nurses in the nursery were trained in its use. However, for the purpose of the study, 5 nurses performed all TcB measurements. We obtained all blood specimens by heel stick. We collected the blood by drip method into heparin-containing tubes. We sent the blood specimen to the laboratory immediately for analysis using Diazo method (Dimension Vist® System and Flex® reagent cartridge, Siemens) [12].

We recorded the following demographic data for all newborns enrolled in the study: gestational age, mode of delivery, sex, birth weight, etiology of jaundice, and postnatal age in hours at the time of TSB and TcB measurements.

### 2.3. Statistical Analysis

We used the Statistical Package for the Social Sciences (version 21, Chicago, IL, USA) for statistical analysis. We used Pearson's correlation and linear regression model including 95% confidence interval (CI) to assess the agreement between TSB and TcB before and during phototherapy. Pearson's coefficient alone can be a poor indicator to estimate the agreement between two diagnostic tests, so we used the Bland-Altman analysis to assess TSB and TcB variability [12]. In this analysis, the mean bias of TCB – TSB was compared against their mean for every patient and variability was defined as ±1.96 standard deviation of the mean bias. Because some studies reported that TcB underestimates TSB at levels >214 *μ*mol/L, we calculated the mean biases before and during phototherapy at TSB levels <214 *μ*mol/L and >214 *μ*mol/L.

## 3. Results

**Figure 1:**
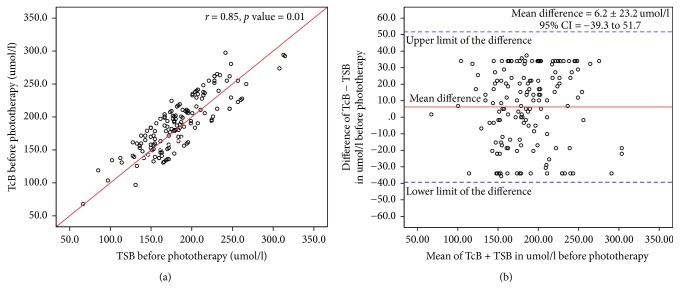
(a) Relationship between transcutaneous bilirubin (TcB) and total serum bilirubin (TSB) levels before starting phototherapy. (b) Bland-Atman Plot of transcutaneous bilirubin and total serum bilirubin values before starting phototherapy.

**Figure 2:**
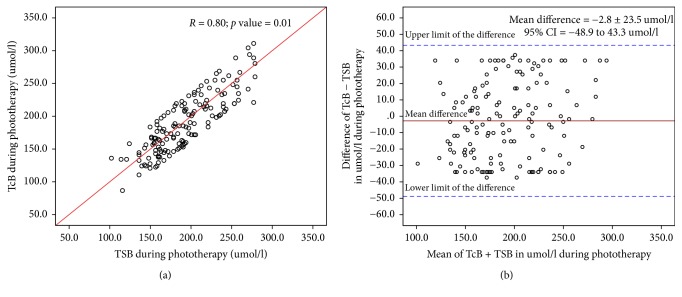
(a) Relationships between transcutaneous bilirubin (TcB) and total serum bilirubin (TSB) levels during phototherapy. (b) Bland-Atman Plot of transcutaneous bilirubin and total serum bilirubin values during phototherapy.

We performed TSB and TcB measurements before and during phototherapy in 151 healthy term infants. The mean gestational age was 38.8 ± 1.2 weeks (range; 37–41 weeks), mean birth weight (BW) was 3.1 ± 0.6 kg (range 1.8–5.2 kg), 80 (53%) were male, and 116 (77%) were born by spontaneous vaginal delivery. The causes of jaundice were physiological jaundice in 62 (41%) newborns, ABO incompatibility in 45 (30%), Rh incompatibility in 10 (6.6%), G6PD in 14 (9.3%), polycythemia in 13 (8.6%), and cephalohematoma in 7 (4.6%). The means of TSB, TcB, and difference between TcB and TSB are shown in Table 1. Linear regression analysis showed a significant correlation between TcB and TSB before starting phototherapy (Figure 1(a)) and during phototherapy (Figure 2(a)) (*r*: 0.85; *p* < 0.001, and *r*: 0.80; *p* < 0.001), respectively. The Bland-Altman plot demonstrates the level of precision of the BiliChek device by comparing the difference versus the average of measurements between TcB and TSB values before (Figure 1(b)) and during (Figure 2(b)) phototherapy. Before starting phototherapy, the mean difference between these measurements was 6.2 ± 23.2 *μ*mol/l with a 95% CI of −39.3 to 51.7 *μ*mol/l. During phototherapy, the mean difference was −2.8 ± 23.5 *μ*mol/l with a 95% CI of −48.9 to 43.3 *μ*mol/l. During phototherapy, TcB measurements underestimated TSB in 53 (35%) infants. TcB underestimated TSB by ≤25.5 *μ*mol/l in 33 (21.8%) infants, by >25.5–≤34 *μ*mol/l in 19 (12.5%) infants, and by >34–51 *μ*mol/l in 1 (0.7%) infant. TSB levels were <214 *μ*mol/L in 111 (73%) newborns. There was a significant correlation between TcB and TSB before starting phototherapy and during phototherapy (*r* = 0.87; *p* < 0.01, and *r* = 0.82; *p* < 0.01), respectively. Before starting phototherapy, the mean difference between TSB and TcB was 10.4 ± 28.1 *μ*mol/l, 95% CI = −44.7–65.5 *μ*mol/l. During phototherapy, the mean difference between TSB and TcB was −3.7 ± 29.3 *μ*mol/l, 95% CI = −60.9–53 *μ*mol/l. TSB levels were >214 *μ*mol/L in 40 (27%) newborns. There was a significant correlation between TcB and TSB before starting phototherapy and during phototherapy (*r* = 0.80; *p* < 0.02, and *r* = 0.77; *p* < 0.04), respectively. Before starting phototherapy, the mean difference between these measurements was −4.5 ± 39.5 *μ*mol/l, 95% CI = −81.9–72.9 *μ*mol/l. During phototherapy, the mean difference between these measurements was −3.6 ± 37.4 *μ*mol/l, 95% CI = −76.9–69.7 *μ*mol/l.

## 4. Discussion

Our study showed a good correlation between TcB as measured from a shielded area from phototherapy at the forehead and TSB measurements in a cohort of jaundiced term newborns during phototherapy. The correlations of TcB and TSB before and during phototherapy were high and comparable. There was no significant difference between TSB and TcB from covered area of the skin. The correlation between TSB and TcB was significant before and during phototherapy when TSB levels were >214 *μ*mol/l, but less than that when TSB levels < 214 *μ*mol/l. The Bland-Atman analysis demonstrated that TcB measurements during phototherapy underestimate TSB (negative mean bias) regardless of TSB levels and there was a wide limit of agreement between TcB and TSB, which worsened as TSB levels increase. In contrast, TcB measurements before starting phototherapy overestimate TSB. The underestimation of TcB to TSB levels by >25.5–51 *μ*mol/l in 13.2% of the studied infants led us to recommend that while the infant is under phototherapy a confirmatory TSB measurement should be done when TcB level is ≤51 *μ*mol/L below the bilirubin level at which phototherapy should be discontinued. Reyes et al. [6] reported that TcB measured by BiliChek during phototherapy compared to TSB has a negative mean bias which worsened as TSB increased particularly at TSB levels > 238 *μ*mol/l. Katayama et al. [13] reported moderate correlations between TSB and TcB before (*r* = 0.56) and during (*r* = 0.47) phototherapy. For TSB ≤ 306 *μ*mol/L during phototherapy, a TcB cut-off of 238 *μ*mol/l had a specificity of 100%. Zecca et al. [9] and Fonseca et al. [10] reported a good agreement between TSB and TcB measured from patched area of the skin, while TcB measurement from unpatched skin underestimates TSB levels. However, Murli et al. [14] found poor agreement between TcB and STB before and during phototherapy in late preterm and term newborns. Different study populations, inclusion of preterm and term newborns in some studies, and different irradiance of phototherapy light can explain these variations in the results of these studies.

TcB measurement in jaundiced, healthy otherwise, full term infants under phototherapy from properly shielded area of the skin is relatively accurate and can be performed to monitor bilirubin levels. It will lead to a decrease in the frequency of painful blood sampling. However, TcB measurement should not be considered a surrogate for the gold standard TSB measurement. If the infant is found to have significant hyperbilirubinemia, a confirmatory TSB should be considered.

The strength of our study includes a prospective study with a relatively good sample size. We included only homogeneous healthy, term infants rather than including mixed population of term and preterm infants. However, our study has some limitations. First, we obtained TcB during phototherapy at 8 ± 2 hours after starting phototherapy, so we do not know if the same correlation between TSB and TcB will exist if we measure TcB during longer intervals, while the infants are still under phototherapy. Second, trained nurses performed measurements of TcB rather than a specific person whether a physician or nurse and this raises the issue of user variability. However, in practice, nurses perform TcB measurements. As with any point of care test, assessment of the competency of the personnel using TcB device is very important.

Future studies should address the correlation of TcB and TSB at specific intervals during the entire time of phototherapy rather than one paired TcB and TSB measurement. These studies should also address the number of painful heel sticks which will be reduced when TcB device is used to monitor the bilirubin levels during phototherapy treatment as well as the cost effectiveness.

In conclusion, our study supports previous studies which concluded that TcB measurements taken from a covered area of the skin in jaundiced, healthy, full term infants under phototherapy correlate significantly with TSB and can be used to monitor bilirubin levels during phototherapy.

## Figures and Tables

**Table 1:** Total serum and transcutaneous bilirubin levels before and during phototherapy in umol/l.

Bilirubin	Mean ± SD	Range
TSB before phototherapy	183.8 ± 41.6	152–314.5
TcB before phototherapy	190.5 ± 43	147–296
Mean	187 ± 32	175–300
Difference	6.2 ± 23.2^*∗*^	−5.2–51
TSB during phototherapy	191.8 ± 39.4	177–278.8
TcB during phototherapy	187.8 ± 45.3	160–311.1
Mean	186.9 ± 44.9	170–287.3
Difference	−2.8 ± 23.5^*∗*^	−86.7–85

^*∗*^ *p* value = 0.2.
